# Understanding the relationship between income and mental health among 16- to 24-year-olds: Analysis of 10 waves (2009–2020) of Understanding Society to enable modelling of income interventions

**DOI:** 10.1371/journal.pone.0279845

**Published:** 2023-02-28

**Authors:** Fiorella Parra-Mujica, Elliott Johnson, Howard Reed, Richard Cookson, Matthew Johnson

**Affiliations:** 1 Health Economics Research Centre, University of Oxford, Oxford, United Kingdom; 2 Social Sciences, Northumbria University, Newcastle upon Tyne, United Kingdom; 3 Landman Economics, Colchester, United Kingdom; 4 Centre for Health Economics, University of York, York, United Kingdom; Utrecht University: Universiteit Utrecht, NETHERLANDS

## Abstract

A substantial body of evidence suggests that young people, including those at the crucial transition points between 16 and 24, now face severe mental health challenges. In this article, we analyse data from 10 waves of a major UK longitudinal household cohort study, Understanding Society, to examine the relationship between income and anxiety and depression among 16- to 24-year-olds. Using random effects logistic regression (Model 1) allowing for whether the individual was depressed in the previous period as well as sex, age, ethnicity, whether the individual was born in the UK, region, rurality, highest qualification, marital status, employment status and attrition, we find a significant and inversely monotonic adjusted association between average net equivalised household income quintiles and clinical threshold levels of depressive symptoms SF-12 Mental Component Summary (MCS score ≤45.6). This means that being in a higher income group is associated with a reduced likelihood of clinically significant depressive symptoms, allowing for observable confounding variables. Using a ‘within-between’ model (Model 2), we find that apart from among those with the very highest incomes, increases in average net equivalised household income over the course of childhood and adolescence are significantly associated with reduced symptoms of anxiety and depression as measured by a higher SF-12 MCS score. Compared with previous reviews, the data presented here provides an estimate of the magnitude of effect that helps facilitate microsimulation modelling of impact on anxiety and depression from changes in socioeconomic circumstances. This enables a more detailed and complete understanding of the types of socioeconomic intervention that might begin to address some of the causes of youth mental health problems.

## Introduction

**Fig 1:**
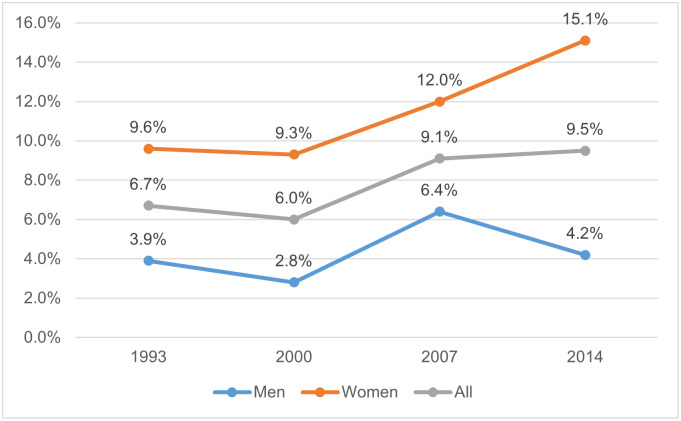
Prevalence of CIS-R scores of 18 or above among 16- to 24-year-olds between 1993 and 2014.

Longitudinal data indicates that young people in the UK face severe mental health challenges. England’s Adult Psychiatric Morbidity Survey (APMS) uses the Clinical Interview Schedule-Revised (CIS-R) measure, with a score of 18 or more indicating severe symptoms of common mental disorders (CMD) that would almost certainly benefit from intervention and treatment. As shown in Fig 1, the proportion of 16- to 24-year-olds with a score of 18+ actually fell between 1993 (6.7%) and 2000 (6.0%), before increasing sharply to 9.1% in 2007 and 9.5% in 2014 [1 Table 2.2]. A large proportion of this increase is seen among women, with prevalence increasing from 9.3% in 2000, to 12.0% in 2007 and 15.1% in 2014 [1 Table 2.2].

Reported rates of self-harm (5.3% to 13.7%) and attempted suicide (1.3% to 2.2%) also increased from 2000 to 2014 among 16-24s in the same surveys [2 p7]. This trend of worsening mental health appears not to have improved since then, with an increase of 5.0 percentage points in probable mental disorders among 11-16s between 2017 and 2020 [2 p6]. In terms of reactive healthcare impact, there were 420,314 open referrals in England to child and adolescent mental health services (CAMHS) in February 2022 [3], a 54% increase since the same month in 2020 [4].

At international level, there is evidence from the Global Burden of Disease Study of increasing prevalence of mental disorders among young people aged 10–24 in European countries between 1990 and 2019 [5], though with a large decrease in incidence of self-harm. There are, though, substantial limitations in the data, with large uncertainties for certain disorders, inconsistent classification and large differences between countries. These between-country differences can be observed in other studies. For example, Buli et al. [6] found that Sweden appears not to have experienced worsening trends in mental health between 2004 and 2020. However, they also found an apparent increase in the adolescent mental health social gradient due to improving trends among higher SES young people [6]. In sum, while there appears to be a trend of worsening adolescent mental health in England, and in many other developed nations, over the last few decades, the data is imperfect.

Social determinants may explain some of these trends, with evidence suggesting a significant diminution in young people’s perceived control over their lives [7]. The PISA 2018 results [8] found that levels of life satisfaction among UK 15-year-olds were second-bottom among the 30 OECD nations included in the analysis, while they also expressed among the lowest levels of agreement with statements related to meaning in life. There are many plausible explanations for this: the 2007/2008 Global Financial Crisis, subsequent austerity measures, Brexit, the COVID-19 Pandemic and, now, the cost-of-living and environmental crises. Indeed, central estimates of the rate of relative child poverty after housing costs in the UK increased from 27% in 2010/11 to 31% in 2019/20 [9 Table 1_4a] and the number of people aged 16–24 in employment remained lower in June-August 2022 than the pre-pandemic quarter of January to March 2020 due to an increase in economic inactivity [10 p4].

Some of this study’s authors have argued elsewhere that Universal Basic Income (UBI) may serve to mitigate these social determinants of health [11, 12]. Indeed, their model of impact [12 p411] sets out the likely causal pathways between welfare systems, UBI specifically, and health outcomes via pathways of poverty reduction, inequality reduction and predictability and security of income which are likely to result in improved satisfaction of material needs, behavioural change that encourages longer-term investment in health and stress reduction. While there is a significant body of evidence to suggest a relationship between income and mental health (see ‘Income and anxiety and depression’ below), the evidence base on UBI is limited in the absence of experimental trials that are designed to measure health impact in the UK and are representative of the kinds of policies that might be implemented. Moreover, microsimulation of impacts has been restricted by the absence of publicly available relative risks or risk differences data by household income that is representative of a UK context, even, for example, in Romero et al.’s [13] systematic review of the impact of cash transfers on mental health and wellbeing. With policymakers increasingly concerned with the need for prevention and a Welsh Government [14] trial of basic income for Care Leavers that began in July 2022, there is need to understand the prospective scale and pathways of impact, particularly among adolescents.

In addition to an existing policy focus on the age group, in part due to the rapidly increasing CAMHS caseload, there is good reason to examine 16-24s as an age group. There is evidence that half of all lifetime cases of mental health conditions emerge by age 14 and three quarters by 24 [15]. Further, McGorry et al. [16 pS5] claim that these youth mental health problems are associated with ‘enduring disability, including school failure, impaired or unstable employment, and poor family and social functioning, leading to spirals of dysfunction and disadvantage that are difficult to reverse’.

This article analyses data from 10 waves (2009–2020) of the UK Household Longitudinal Study (UKLHS), also known as Understanding Society. We first use a random-effects logit model (Model 1) by net equivalised household income to provide estimates of magnitude that would enable microsimulation of upstream income interventions. Understanding Society’s definition of a household follows

UK statistical practice and is the same as that used in UK government surveys. A household is ‘one person living alone or a group of people who either share living accommodation or share one meal a day and who have the address as their only or main residence’. The definition also requires six months’ continuous residence, implying that students will be included at their term time address, unless living at a hall of residence.

[17 p9]

We then use a ‘within-between’ model (Model 2) to analyse the associations between the SF-12 Mental Component Score and: i) between-individual differences in income, and ii) within-individual variations of income across time. We model non-linearity in, and interactions between, these within- and between-effects (Models 2a and 2b). The comparison between units enables further examination of the lifestyle factors that affect the relationship between income and mental health within the 16- to 24-year-old age group. We discuss the implications of those factors on policymaking, arguing that the results support an upstream intervention with low levels of conditionality, such as UBI. We begin by outlining the existing evidence on income and anxiety and depression.

### Income and anxiety and depression

The relationship between income, inequality and mental health has been investigated through a range of systematic reviews and individual studies [18], identifying associations in a range of demographic groups and populations. While most identified at least reasonable evidence of an association, Thomson et al.’s [19] review highlighted the variance in quality of data and evidence. Cooper and Stewart [20 p981], however, examined studies that lend themselves to causal interpretation (i.e., Randomised Controlled Trials, quasi-experiments and fixed effect-style techniques on longitudinal data) and found that ‘results lend strong support to the hypothesis that household income has a positive causal effect on children’s outcomes, including their cognitive and social-behavioural development and their health, particularly in households with low income to begin with’. Importantly, they conclude that ‘effects tend to be larger in experimental and quasi-experimental studies than in fixed effect approaches’ [20 p981], suggesting that analyses from observational studies looking at a range of children’s outcomes (as well as maternal depression) may produce conservative estimates of associations. Benzeval et al. [21] summarised the state of play, finding a ‘strong theoretical consensus that money does matter for health and the relationship is a positive one’, but that there is ‘less clarity regarding the particular role of income as a health determinant or the mechanisms by which income modification interventions might affect health’ [21 p52].

Clear evidence on the impact of income and income changes on adolescent and early adulthood anxiety and depression is essential both because young people are facing apparent increasing rates of mental health problems and there is increasing interest among policymakers of the possibility of addressing health through socioeconomic interventions. From an economic perspective, even in pre-pandemic 2019, 26% of 18- to 29-year-olds in work said that they struggle to make ends meet financially [22 p9]. In addition, the National Living Wage is only available to those aged 23+ with decreasing minimum wage rates for each age group under 23 [23]. In terms of benefits, many have lower rates for those aged under 25 [24]. In general, with a few exceptions, benefits are not available to under-18s on account of their being expected to be dependent on family or carers [25 p21]. Alongside this, the percentage of young people aged 21–24 living with parents has increased from 41.7% in 2006 (almost exactly the same as 10 years previously) [26] to 54.7% in 2021 [27 Table 2021], delaying independent living and the autonomy that brings (see Nussbaum [28]). Indeed, with graduate opportunities now declining, people’s wellbeing within the cohort is reduced and their ability to transition into adulthood is, consequently, diminished. The effect of diminished opportunity for income and independence is compounded by inequality. In broader economic terms, using Gini coefficient data from 2017–2020, the UK has the sixth-highest income inequality among OECD nations with available data [29]. Pickett and Wilkinson [30] have highlighted the significant correlations between income inequality and worse child mental health outcomes.

Increasingly, there is emphasis in healthcare on preventive intervention, rather than solely reactive treatment. While Patton et al. [31 p37] found that intervention in adolescence may help to avoid early experiences of mental health conditions continuing or recurring in adulthood, the inability of purely reactive, clinical ‘healthcare’ interventions to address mental health conditions has been highlighted by Stockings et al.’s [32] meta-analysis. This found that the efficacy of such interventions in relation to both depression and anxiety among children and adolescents aged 5–18 was only short-term in nature, while suggesting that repeated exposures should be considered. As such, there is a need to develop upstream interventions that have long-lasting impacts on young people’s mental health that persist into adulthood.

There have been calls to trial UBI as an upstream intervention to mitigate poverty, inequality and insecurity as social determinants of anxiety and depression. However, in the absence of a randomised controlled trial (RCT) in the UK, it is necessary to inform policymaking by analysing longitudinal datasets to produce relative risk factors by which to conduct modelling. This article presents those risk factors and attempts to examine the reasons for the distribution of those risks.

In doing so, we seek to build on existing evidence of association between income and mental health to answer two related questions:

1. What is the magnitude and shape of association between **average** household income and mental health among 16-24s?
2. What is the magnitude and shape of association between **changes** in household income and mental health among 16-24s?

## Methods

### Data

This article uses the panel data available in 10 waves of the UK Household Longitudinal Study (UKLHS), which is also known as Understanding Society. The waves began in January of each year between 2009 and 2018 and ended between March and June two calendar years later (2011–2020) [33]. The UKHLS draws upon interview data from 40,000 households to present socioeconomic, demographic and health data of individuals living in private households in the UK. It is an extension of the British Household Panel Survey (BHPS) which ran from 1991 to 2009. Participants in Understanding Society are interviewed each year and the study covers all members in the household. One person in the household completes a household questionnaire (including questions on household income), and everyone in the household aged 16 and over completes an adult questionnaire which covers a range of dimensions, including mental health.

The Ethnic Minority Boost Sample [34] design enables the analysis of ethnicity and comparison of individual ethnic minority groups by including at least 1,000 adults from each of five target ethnic minority groups (Indians, Pakistanis, Bangladeshis, Caribbeans and Africans) within the main equal probability sample. The boost sample was designed around variations in ethnic densities in small areas within Great Britain. The boost sample design selected an additional sample of households in areas of high density of each of the target groups plus other minority groups.

The main sample of the UKHLS includes around 2,800 adults from the groups covered by the boost sample, plus some others from groups not covered by the boost (for example white minorities). Combining the boost sample with the main sample provides large numbers of members of minority groups for analysis of the full range of survey data.

We used the longitudinal weight variable ‘indinus’ for analysis of individual interview data from multiple waves between waves 1 and 10 for the primary sample and the Ethnic Minority Boost Sample combined. The purpose of this was to correct for unequal selection probability, lack of response in the first wave, and attrition at following waves. Additionally, we use attrition controls in our regressions to correct for nonresponse, as differences in selection and response likelihood affect the relationship between mental health and other independent variables (such as income) in the models.

With regard to attrition controls, we create a next-wave dummy (if the individual participates in the survey the following year), an all-wave dummy (if the individual has participated in all subsequent waves after starting) and a variable counting the number of times the individual has participated in the survey to account for the effect of survivorship bias. As Contoyannis, Jones and Rice [35] suggest, there is attrition from the panel at each wave and some could be due to health issues (deaths, serious illness, participants moving to care homes). This means that the long-term participants of the survey are more likely to be healthier, on average, than those who dropped out. If we do not take into account this attrition, we might fail to correctly measure the dynamics between health and socioeconomic characteristics. Indeed, Contoyannis, Jones and Rice [35] use the British Household Panel Survey (the predecessor of the UKHLS) to show that attrition rates are inversely related to initial health and that attrition is highest among those who start the survey in very poor health, those with lower incomes and those with less formal education and is particularly high among those who had never married at the start of the panel. S2 Stata includes the data file used to undertake all analysis.

Table 1 summarises the main variables used in the model: income, demographics and health outcomes. The total analytical sample size is 9,581 individuals and 24,248 observations. We included anyone in the panel survey who was between the ages of 16 and 24 (inclusive) from 2009/10 to 2019/20. Most individuals are observed repeatedly over several years, but this might not always be the case: for instance, there are those who only entered the sample frame once in the first or last year (i.e., those who were 24 in 2009/10 and those who were 16 in 2019/20). In the case of individuals who were aged 16 in 2009/10, they were observed 10 times over 10 years in this sample frame, but all others were observed fewer than 10 times.

**Table 1:** Descriptive statistics for Understanding Society 16–24 SF-12 Mental Component Summary sample.

**Individuals**	9,581
**Observations**	24,248
	**Mean**
**SF-12 MCS score**	47.35
**Age**	20.23
**Income**	
Net household income (non-equivalised, monthly mean)	£3,666.60
Net equivalised household income (monthly mean)	£1,577.59
Net equivalised household savings (monthly mean)	£77.26
Household size (mean)	3.79
Average number of OECD children per household	0.40
**Gender**	**Percentage**
Female	55.17%
Male	44.83%
**Ethnicity** *	
White British/English/Scottish/Welsh/Northern Irish	74.49%
White (other)*	3.08%
Mixed**	3.71%
South Asian (Indian, Pakistani, Bangladeshi)	11.54%
Chinese and any other Asian background	1.46%
Caribbean, African and any other black background	4.85%
Other	0.86%
**Country of birth**	
Born in the UK	91.93%
Not born in the UK	8.07%
**Marital status**	
Single	87.29%
Cohabiting	9.80%
Married/in a civil partnership	2.73%
Divorced	0.09%
Other	0.09%
**Occupational classification (NS-SEC)**	
Managerial and professional	44.33%
Intermediate	9.73%
Small employers and self-employed	2.79%
Lower supervisory and technical	42.02%
Semi-routine and routine	1.14%
Never employed	21.15%
**Labour market status**	
Employed	44.33%
Unemployed	9.73%
Family care	2.79%
Full-time student	42.02%
Other	1.14%
**Education**	
No Qualification	2.23%
GCSE or other qualification	27.52%
A Level	49.09%
Higher degree / University	21.15%
**Household tenure**	
Own (outright or with mortgage)	60.69%
Renting or other	39.33%
Individuals per room (average)	0.78

* Uses ‘racel’ variable

** Includes: Irish, gypsy or Irish traveller, and any other white background

*** Includes: white and black Caribbean, white and black African, white and Asian, and any other mixed background

### Measures

General variables in the analysis include the equity-relevant characteristics: sex, age, ethnic group, and whether or not the individual was born in the UK. A full list of variables used can be found in S1 Table.

#### Mental health

Among other measures, participants reported their mental health using the Mental Component Summary of the Short-Form Health Survey (SF-12) [36], which has been used to screen for general, non-psychotic mental health problems among primary care patients. A score of ≤45.6 on the SF-12 MCS indicates clinical level depressive symptoms [37]. We use SF-12 MCS score as outcome variables for our within-between model. The items comprising SF-12 can be found in the Understanding Society Wave 10 questionnaire [38 pp560-564]. The scoring system used can be found in Ware et al. [39]. Additionally, we create a dichotomous variable depression which takes the value of 1 if the individual scores ≤45.6 and 0 if they score above.

#### Socioeconomic units

Net equivalised household income is the sum of net monthly incomes from all household members, adjusted by the OECD-modified equivalence scale [40] to account for households of different size and composition. The results were deflated using the Consumer Price Index (excluding housing costs), to express income in January 2015 prices.

### Analyses

Our analysis is two-fold. First, we use a logistic regression (Model 1) to model the probability of an individual scoring below clinical levels of mental health symptoms, given different income quintiles. That way, we were able to compare the highest income, with all other less advantaged groups, reporting the magnitude of the predicted probability and statistical significance. Second, we use a ‘within-between’ model (Model 2) to analyse the associations between SF-12 MCS score and: i) between-individual differences in SES, and ii) within-individual variations of income across time.

The specific model designs are further described below:

#### Random effects logit model

We model the probability of a discrete binary outcome (scoring ≤45.6—indicative of clinical levels of depressive symptoms—or ≥45.7) given the input variable income quintile and control variables including sex, age, ethnicity, region, rurality, whether or not the individual was born in the UK, marital status, employment status and an attrition-based control. To address potential reverse causation bias, we also control for whether the individual was depressed in the previous period, as explained below. The modelled association between income and the probability of depressive symptoms is thus adjusted for the values of the covariates. We propose the following model:

### Model 1


$$\text{ln}\left(\frac{P(h_{it}=1|x_{it},u_{it})}{P(h_{it}=0|x_{it},u_{it})}=1\right)=\alpha_{i}+\overset{n_{i}}{\underset{i=1}{\sum }}\beta_{1}x_{it}+\;u_{it}$$

Where *h*_*it*_ is the binary outcome, *α*_*i*_ is an individual-specific and time-invariant random effect, *β*_1_ is a vector of regression coefficients including both control variables and income quintile dummy variables, and *u*_*it*_ is the error term, uncorrelated across individuals and over time for one same individual. This model was obtained from Wolf [41].

Our results are presented as adjusted marginal effects, or differences in probabilities, which are more easily interpretable than log odds.

#### ‘Within-between’ regressions

We examined the impact of changes in household income throughout childhood and adolescence on changes in mental health. This is important because a) changes in income may result in changes in lifestyle that children might be more aware of and b) changes in income provide a proxy for the kinds of socioeconomic impact that cash transfers, like those under UBI, might have.

We used a ‘within-between’ model, with lagged health outcomes, to disentangle the relationship between mental health and: i) between-individual differences in income, and ii) within-individual variations of income across time. The ‘within-between’ linear model is a reformulation of the Mundlak model and has a significant advantage in being able to retain the flexibility of random effects models while reducing concerns about bias that fixed effects models address [42, 43].

### Model 2


$$h_{it}=\beta \prime \bar{Income_{\dot{i}}}+\gamma^{\prime }(Income_{it}-\;\bar{Income_{\dot{i}})}+\;\delta^{\prime }h_{it-1}+\;\eta \prime c_{i}+\left(u_{i}+e_{it}\right)$$

Where *h*_*it*_ is the dependent variable, the health outcome, for individual *i* at time *t*. *x*_*it*_ is the set of observed variables of interest, separated into: ${\overset{-}{x}}_{i}$, individual *i*’s average over the sample period, and ($x_{it}-\;{\overset{-}{x}}_{i})$, *i*’s difference from their average at time *t*. Variables include all time-varying observable variables: our main SES variable of interest, alongside time varying demographic controls (age, age squared, marital status).

The corresponding coefficients are *β*, the ‘between-effects’, and *γ*, the ‘within-effects’. Lagged health variables are represented by *h*_*it*−1_, while *c*_*i*_ are the time-invariant control variables, such as demographic controls (sex, ethnicity, region, rurality, whether or not the individual was born in the UK), initial health states (*h*_*i*1_) and attrition controls (wave count and all waves), with *δ* and *η* as the corresponding coefficients. The residuals *u*_*i*_ and *e*_*it*_ are assumed to have a mean of zero and be normally distributed.

Intuitively, this approach allows the identification of the ‘between’ effects, as the effect of differences in income between persons, and the ‘within’ effects, as the effect of an increase/decrease in an individual’s income relative to their average income. Furthermore, the inclusion of a lagged health variable is an attempt to reduce the impact that reverse causality has on the estimates. Equation 1 controls for the previous years and the ‘initial’ state of mental health.

We then added quadratic terms to identify non-linearities in the relationships between health and between-individual differences in average income and within-individual increases in income. The first model incorporates a quadratic term for both within and between effects:

### Model 2a: Quadratic model


$$h_{it}=\beta^{\prime \bar{x_{\dot{i}}}}+\beta_{A}\prime {\bar{x}}_{i}^{2}+\gamma^{\prime }\left(x_{it}-{\bar{x}}_{i}\right)+\gamma_{A}\prime \left(x_{it}^{2}-{\bar{x}}_{i}^{2}\right)+\delta^{\prime }h_{it-1}+\;\eta \prime c_{i}+\left(u_{i}+e_{it}\right)$$

Coefficients *β*_*A*_ and *γ*_*A*_ capture the quadratic terms for the between and within effects respectively. This uncovers differential marginal effects of an increase in income.

The second model, allows for an interaction between the two levels:

### Model 2b: Interaction model


$$h_{it}=\beta^{\prime }{\bar{x}}_{i}+\gamma^{\prime }\left(x_{it}-{\bar{x}}_{i}\right)+\gamma_{B}\prime \bar{x_{i}}\left(x_{it}-{\bar{x}}_{i}\right)+\delta^{\prime }h_{it-1}+\;\eta \prime c_{i}+\left(u_{i}+e_{it}\right)$$

This allows us to identify if a within-individual increase in income depends on their average level of income. In other words: does an increase in within-individual income for someone with lower average income have a greater impact than on someone with higher average income?

- *h*_*it*_: health outcome, for individual *i* at time *t*.
- $\bar{Income_{i}}$: individual *i*’s average over the sample period.
- $\left({Income}_{it}-\bar{Income_{i}}\right)$: individual *i*’s difference from their average income.
- *β*: ‘between effects’; *γ*: ‘within effects’.
- *h*_*it*−1_ (lagged health variables) and *c*_*i*_ (time-invariant variables) are control variables.
- *u*_*i*_ and *e*_*it*_ are the residuals.

#### Addressing reverse causation basis

**Fig 2:**
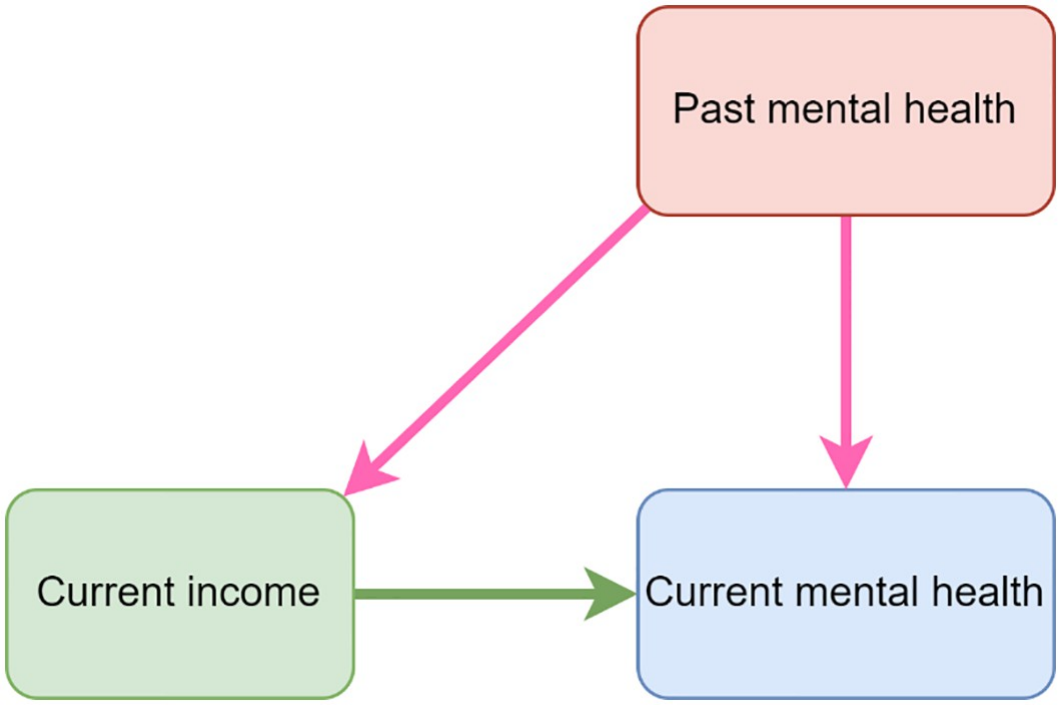
DAG diagram of the relationship between current income, past mental health and current health.

While reviews find correlation between income and mental health, there has been debate about the direction of causation. Mental health clearly has the potential to impact income and employment. The Health Foundation has recently [44] concluded that the relationship is bi-directional. Reverse causation bias might emerge if year-on-year change in mental health causes year-on-year change in household income by influencing employment and earnings. This might lead to an over-estimate of the causal effect of household income on mental health. However, we address this potential bias by controlling for last year’s mental health. Our DAG diagram (Fig 2) illustrates this through the pink causal pathways. This a standard strategy in health econometrics and is used and justified, for example, by Contoyannis, Jones and Rice [35]. However, it is arguably conservative in focusing on the year-on-year effect rather than the cumulative long-term effect.

This strategy also integrates potentially unmeasured effects in the association, for example effects of past physical health on past mental health. It assumes that mental health effects on employment and earnings usually occur with a lag of about a year; that is, they do not usually occur within our measured time period of one year. If effects of mental health on earnings usually occur much more rapidly than this, then our model could still be subject to reverse causation bias: a decline in mental health early in the current year could still reduce income this year.

Furthermore, from ages 16 to 24, a substantial part of household income derives from parental employment and earnings as well as, for many, an individual’s own employment and earnings. There may be a causal effect of own past mental health on current parental employment and earnings, but this is likely to be a second order effect compared with the effect on individual employment and earnings.

It is possible to run panel data models with more complicated lag structures, known in econometrics as dynamic panel models, of which cross-lag models are a special case. However, given various well-known statistical difficulties with such models (see, e.g., Hamaker, Kuiper and Grasman [45]), there is little reason to conclude that more complicated modelling strategies would yield a substantially more credible estimate in this case. We therefore believe that the risk factors developed represent the best estimate obtainable from observational data, in the absence of a directly relevant randomised controlled trial or natural experiment.

## Results

### Descriptive statistics

Table 1 provides descriptive statistics of the Understanding Society longitudinal SF-12 analytical sample.

**Fig 3:**
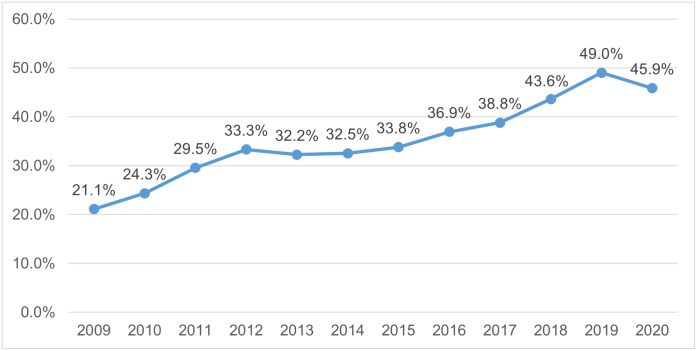
Percentage of respondents with an SF-12 MCS score of ≤45.6 indicating clinical depressive disorder by year of interview.

Table 2 and Fig 3 show the number and percentage of respondents with an SF-12 MCS of ≤45.6, the clinical threshold score for depressive disorders, by the year they were interviewed within waves 1–10. We observe that the proportion of participants meeting the clinical threshold score rises significantly over time. We believe that this reflects a genuine trend of increasing rates of depression among young people, as highlighted in the Introduction. However, there may also be some sampling issues at play, in spite of the likelihood that attrition would lead to healthier participants, on average, remaining in the study.

**Table 2:** Number and percentage of respondents with an SF-12 MCS of ≤45.6 indicating clinical depressive disorder by year of interview.

Year of interview	Not depressed (%)	Depressed (%)	Total number
2009	78.9%	21.1%	3,574
2010	75.7%	24.3%	7,603
2011	70.5%	29.5%	7,299
2012	66.7%	33.3%	6,561
2013	67.8%	32.2%	6,309
2014	67.5%	32.5%	5,907
2015	66.2%	33.8%	5,792
2016	63.1%	36.9%	5,424
2017	61.2%	38.8%	4,591
2018	56.4%	43.6%	4,143
2019	51.0%	49.0%	2,023
2020	54.1%	45.9%	142
Total	67.0%	33.0%	59,368

### Net equivalised household income

**Fig 4:**
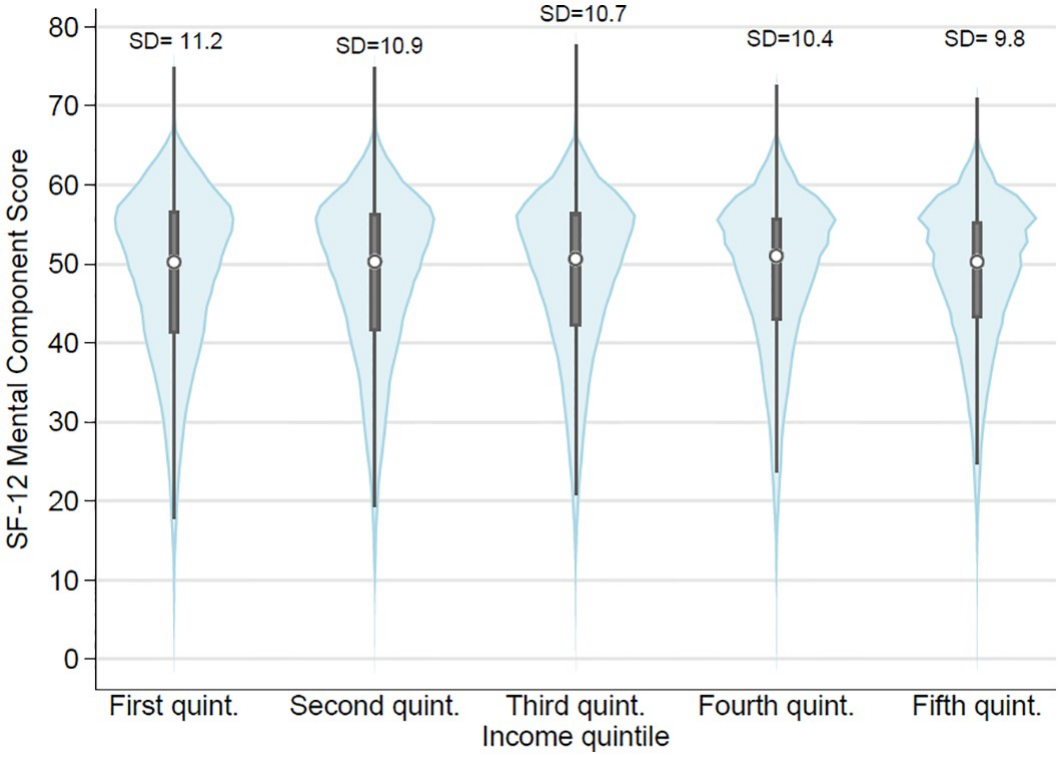
Violin plots of SF-12 MCS scores by net equivalised household income quintiles.

Fig 4 shows violin plots of SF-12 MCS scores across the five quintiles in net equivalised household income distribution. Fig 4 includes all the observations across all waves. Violin plots overlay a plot of the estimated kernel density to the summary statistics displayed by box plots. It includes a marker for the median of the data, a box indicating the interquartile range, and spikes extending to the upper- and lower-adjacent values. The median for all groups varies between 50.2 and 51 and increases in each subsequent income quintile until the fourth then drops slightly to 50.3 in the highest quintile. We observe that individuals from higher income groups have less variation (represented by the standard deviation) compared to individuals from lower income groups, which explains the longer tails that lower income groups display.

#### Probability of scoring SF-12 MCS ≤45.6 indicating clinical depressive disorder by net equivalised household income

We ran a random-effects logistic regression (logit) to estimate the probability of scoring ≤45.6, the SF-12 MCS cut-off to screen for 30-day depressive disorders [37]. We controlled for relevant demographic characteristics including sex, age, region, rurality, ethnic background, born in the UK, marital status, employment status in the previous period, and an attrition-based control. We also controlled for whether or not the individual scored below the cut-off in the previous wave, as well as controlling for the wave number.

Our results are presented as marginal effects, or differences in probabilities, for easier interpretation. Hence, results can be interpreted as the predicted probabilities for individuals in a given income quintile if they were all average in all other respects. Table 3 shows that, in comparison to two otherwise-average individuals, the adjusted prediction of an individual in the lowest income quintile is 0.373. This probability falls to 0.348 in the second quintile and keeps dropping for the subsequently higher quintiles.

**Table 3:** Adjusted prediction of an individual scoring SF-12 MCS ≤45.6 indicating clinical depressive disorder by net equivalised household income quintiles.

Income quintile	Probability of scoring SF-12 MCS ≤45.6	Standard error	P-value	95% confidence interval low	95% confidence interval high
First (lowest)	0.373	0.008	0.000	0.356	0.389
Second	0.348	0.008	0.000	0.333	0.364
Third	0.342	0.008	0.000	0.328	0.357
Fourth	0.324	0.007	0.000	0.310	0.338
Fifth (highest)	0.315	0.007	0.000	0.301	0.329

As the probability of scoring SF-12 MCS ≤45.6 (indicating clinical depressive disorder) decreases among individuals in higher income quintiles, we can infer that there is a negative marginal effect of being in a higher income quintile on the probability of scoring below the threshold. This is confirmed in Table 4, which shows the marginal effect of each quintile on the probability of scoring below the threshold, compared to being in the lowest quintile. For instance, being in the second income quintile reduces the probability of scoring below the depression threshold by 0.024 compared to being in the first quintile. On the other hand, being in the highest (fifth) quintile reduces the probability of scoring below the depression threshold by 0.058 compared to being in the lowest (first) quintile. This evidences the diminishing marginal returns that being in a higher income quintile has on an individual’s mental health score. For instance, if an individual goes from the lowest (first) quintile to the second quintile, this one-quintile change negatively impacts their probability of being depressed in -0.024. If the individual instead upgrades to the third quintile, this two-quintiles change (from the lowest to third quintiles) does not have double the impact of moving from the lowest to second quintile (i.e., 2* -0.024 = -0.048) but a more discrete impact in their probability of being depressed (-0.030).

**Table 4:** Marginal Effects at the Means (MEM) by net equivalised household income quintiles.

Income quintile	Differences in probability of scoring SF-12 MCS ≤45.6	Standard error	P-value	95% confidence interval low	95% confidence interval high
Second	-0.024	0.011	0.026	-0.046	-0.003
Third	-0.030	0.011	0.006	-0.052	-0.009
Fourth	-0.049	0.011	0.000	-0.070	-0.027
Fifth (highest)	-0.058	0.011	0.000	-0.079	-0.036

**Fig 5:**
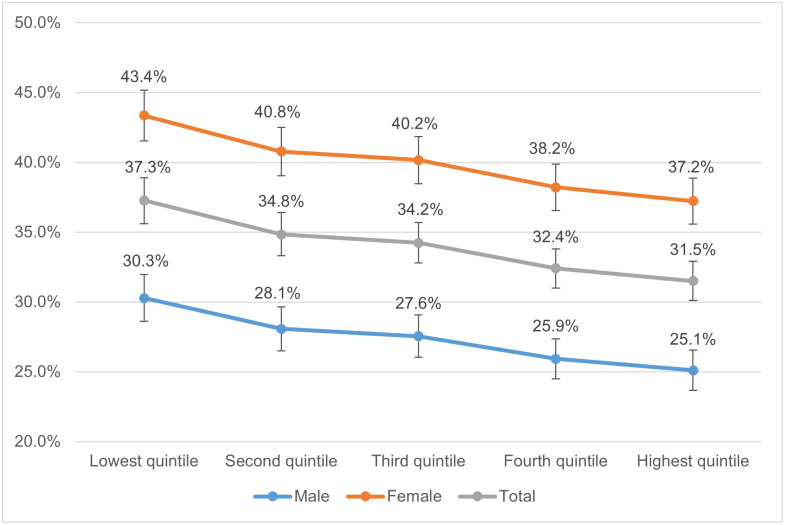
Probability of scoring SF-12 MCS ≤45.6 indicating clinical depressive disorder by net equivalised household income quintiles.

Fig 5 shows that, for both men and women, the probability of scoring SF-12 MCS ≤45.6 indicating clinical depressive disorder decreases for individuals at higher income levels. The marginal effect that being in a higher income quintile has on your probability of scoring below the threshold is significantly higher for the most deprived quintiles. For instance, the marginal effect of being in the second quintile compared to being in the lowest quintile is stronger than the marginal effect of subsequent income increase, as shown by the curve in Fig 5 tending to flatten for the wealthiest quintiles. The shape of the gradient here differs from similar analysis undertaken using Millennium Cohort Study data covering 14- and 17-year-olds [18]. In that analysis, there was a slightly non-monotonic relationship at the lowest and second quintiles and a steeper gradient at the higher income quintiles. That difference could perhaps be due to only one age cohort having been used in the Millennium Cohort Study, observations only taking place at ages 14 and 17, fewer income data points from which to construct the average, and different measures of mental health (SMFQ and Kessler-6) having been used.

#### Within-between model: SF-12 MCS score by net equivalised household income quintile

Table 5 presents results from the ‘within-between’ model (Model 2), with SF-12 MCS score as the outcome variable and the individual’s net equivalised household income quintile as input variable. We controlled for relevant demographic characteristics including sex, age, region, rurality, ethnic background, born in the UK, marital status, and employment status in the previous period. We also controlled for attrition, past mental health and study wave number. Results show that differences between individual’s average levels of income and within individual’s changes of income are both positive and highly significant predictors of mental health. The between effect shows that, keeping all else constant, individual A who is one income quintile above individual B, also has an SF-12 MCS score that is 0.320 points higher on average. This shows a socioeconomic gradient in mental health between individuals of differing average income. The within-effect shows the difference in SF-12 MCS score for an individual whose income increases from one quintile to the next. This association is, on average, 0.133 points. Being female is associated with an SF-12 score that is 1.659 lower, on average. Individuals with a mixed or Chinese and any other Asian ethnic background also show lower SF-12 scores, followed by white individuals.

**Table 5:** Within-between model (Model 2): SF-12 MCS and net equivalised household income quintile.

	Coefficient (SF-12 MCS points)
Between-Income	0.320***
Within-Income	0.133*
**Controls**:	
Sex (Female)	-1.659***
SF-12 Score (T-1)	0.472***
Unemployed (previous period)	-0.111
Urban	-0.308*
Not born in the UK	0.525**
Married (within-individual change)	1.690**
Married (between individuals differences)	-0.001
**Ethnicity**:	
White: British/English/Scottish/Welsh/Northern Irish	-1.207*
White: (other)*	-1.019
Mixed	-1.894**
South Asian (Indian, Pakistani, Bangladeshi)	-0.359
Chinese and any other Asian background	-1.744**
Caribbean, African and any other black background	-0.340
Other	0.000 (Control)
**Region**:	
England	-1.492***
Scotland	-1.374***
Wales	-1.234***
Northern Ireland	0.000
**Observations**	24,179
**Individuals**	9,541
**Log-Likelihood**	(8.441)

* p < 0.10,

** p < 0.05,

*** p < 0.01

Table 6 presents the results from a non-linear version of the within-between model, with SF-12 MCS as the outcome variable and the individual’s household income quintile as input variable along with other controls. These two specifications allow for an additional flexibility: the first (Model 2a), incorporates quadratic terms of the within and between income variables, and the second (Model 2b), allows for the interaction between the within and between levels of income. In Model 2a in Table 6, both the between effect and between effect squared are positive but insignificant. The within-effect suggests an average increase of 0.285 points in the SF-12 MCS score for an individual whose income increases from one quintile to the next, keeping all other variables constant. However, the latter coefficient is insignificant. The within-effect squared is negative but barely insignificant. This would suggest that the association between income quintile and mental health outcomes is not constant at all points along the curve but instead weakens when individuals move from one quintile to a much higher one.

**Table 6:** Within-between quadratic (Model 2a) and interaction (Model 2b) models SF-12 MCS associations by net equivalised household income quintile.

	Model 2a	Model 2b
	Quadratic Coefficient	Interaction Coefficient
Between-Income	0.307	0.326***
Between-Income Sq.	0.005	
Within-Income	0.285	0.194
Within-Income Sq.	-0.037	
Between X Within		-0.0274
**Controls**:		
Sex	-1.671***	-1.671***
SF-12 Score (T-1)	0.472***	0.472***
Unemployed (previous period)	-0.114	-0.112
Urban	-0.303*	-0.303*
Not born in the UK	0.531**	0.531**
Married (within-individual change)	1.688**	1.691**
Married (between individuals differences)	0.000	-0.00125
**Ethnicity**:		
White: British/English/Scottish/Welsh/Northern Irish	-1.227*	-1.229*
White: (other)*	-1.052	-1.055
Mixed	-1.932***	-1.934***
South Asian (Indian, Pakistani, Bangladeshi)	-0.389	-0.390
Chinese and any other Asian background	-1.728**	-1.729**
Caribbean, African and any other black background	-0.334	-0.335
Other	0.000	0
**Region**:		
England	-1.477***	-1.475***
Scotland	-1.354***	-1.353***
Wales	-1.224***	-1.224***
Northern Ireland	0.000	0
**Individuals**	24,179	24,179
**Observations**	9,541	9,541
**Log-Likelihood**	(8.453)	(8.442)

* p < 0.10,

** p < 0.05,

*** p < 0.01

Model 2b shows that within-individual increases in income are strongly associated with better mental health scores, but this association weakens for individuals who had been, on average, in higher income quintiles. There is a non-significant effect of a within-individual increase in income: for an individual who has been (on average) at the lowest quintile but upgrades to the second quintile, keeping all else equal, this income upgrade is associated with an increase of 0.167 in their SF-12 MCS score. The coefficient of the interaction term is negative and significant, suggesting that for individuals that had been (on average) at the higher end of the income distribution, further increases in their income are associated with smaller improvements in their SF-12 MCS score. For instance, if an individual had been, on average, in the fourth quintile of the distribution, and in the next period they are now on the top (fifth) quintile, this income increase would be associated with a 0.084 increase in their SF-12 MCS score. That is, the association is half the size.

## Discussion

Our analysis of 10 waves of Understanding Society data supports the notion of increasing rates of mental health problems among adolescents and young adults in the decade or so between 2009 and 2020 and that there is evidence of underlying social and economic causes. Prevalence of clinical threshold level SF-12 scores appear to increase following the 2010, 2015, 2017 and 2019 General Elections and the 2016 Referendum on EU Membership. Central estimates of relative child poverty after housing costs have also increased between 2010/11 and 2019/20 [9 Table 1_4a] and opportunities for young people to secure stable employment with scope for social mobility and to be protected against negative life events by easily accessible welfare support have reduced. While the overall rate appears high, and may be indicative of a sampling issue, evidence generally indicates that depression and anxiety are indicators of increased rates of attrition [46]. Analysis of distribution of morbidity supports the key findings of Thomson et al.’s [19] systematic review of evidence on the relationship between income and mental health for working-age adults: ‘Effects are potentially larger for wellbeing outcomes, for income losses, and in the most socioeconomically disadvantaged’. The findings provide relative risk data by which to quantify each of those assertions.

The findings also support those of Akanni, Lenhart and Morton’s [47] longitudinal fixed-effects model for health and wellbeing outcomes using Understanding Society data in relation to all ages. They found that household real disposable income positively affects general health, mental health, and life satisfaction. Specifically, they found that an increase in household disposable results in an increased odds ratio of reporting less distress in mental health outcomes using GHQ-12 of about 16.18%. Using several models to estimate cross-sectional regressions of health and wellbeing outcomes using longitudinal income information, they also found that long-term income and income position stability increases the likelihood of reporting improved physical and mental health and increased satisfaction with leisure and life. [47 p7]. This reflects Whitfield, Betancur, Miller and Votruba-Drzal’s [48] findings using data from young adults and their families in the National Longitudinal Study of Youth (NLSY79) and its Young Adult Survey supplement (NLSY-YA). Using a using a modified version of the Center for Epidemiological Studies Depression Scale (CES-D), they found that childhood income volatility predicted higher depression scores in adulthood.

In addition to the issue of reverse causation bias, there are two key aspects of those findings that require further examination.

### Medication vs income

Asserting that the primary direction of causation is from income to mental health need not dismiss either the need for investment in medical interventions or recognition of the impact of mental health on people’s outcomes. Addressing mental health downstream or through medicalisation has the capacity to get some people back into work and improve outcomes. However, there are good sociological reasons, in the UK in particular, to think that the most prevalent direction of causation is from income/wealth to mental health outcome. For mental health status to be the most or only significant driver of population income distribution, society would have to be extremely fluid, with significant increases/decreases associated largely or solely with employment performance. In contrast, the Social Mobility Commission [49 pxv] found that, in the UK, every ‘critical measure of low social mobility—child poverty, income inequality, access to stable housing, unemployment for young people and gaps in school attainment—was poor in 2019’. As a result, opportunities for those with better or improving mental health to take advantage of it economically and to enhance their income are reducing, especially for young people. The cost of radically increasing income through training, acquisition of materials or resources, etc., is historically high (e.g., Reay [50]). As such, people’s income is likely to follow fairly stable trajectories for their own sectors of employment or sources of welfare, with large changes in income the result of significant life events, such as trauma, and serious mental health crises which, again, disproportionately occur at the lower end of the SES spectrum, or outlying luck. For the 16–24 cohort, there is even greater reason to acknowledge that the burden of addressing inequalities should not be on the individuals at the sharp end of their effects. The younger part of the cohort has few or no options with regard to improving the immediate financial situation of their families due to requirements to be in education or training, while the older segment is likely to have been limited in their opportunities to secure qualifications and experience that lead to higher incomes due to the costs inherent in that process. Indeed, the only options individuals in this cohort from lower-income families have are to enter lower-paying work at a younger age, therefore forfeiting higher salaries later, or to remain in full-time education and endure worse immediate material circumstances.

As such, our findings endorse Thomson et al.’s [19] key policy conclusions: ‘To support mental health, welfare policies need to reach the most disadvantaged, and consider wider factors such as financial insecurity and employment’. With the limited exception of those at the higher end of the SES spectrum whose mental health may be affected by financial strain associated with competitive spending overcommitments [51 p36], there is no evidence that an increase in income has an otherwise negative impact on mental health. This may then have a significant subsequent impact on income (with necessary caveats on opportunity, etc.). None of the above suggests that we should not deploy a range of interventions and therapies for existing conditions, but there is good evidence to suggest that increases in income can prevent and treat incidences. Indeed, the evidence we are examining supports the notion of increases in income being the ‘ultimate “multipurpose” policy instrument’ [52 p145].

However, contra Thomson et al.’s [19] concern for the most disadvantaged, the evidence presented here suggests that reform to address insecurity and employment needs to support a much larger cohort than the most disadvantaged. The cost-of-living crisis is likely only to highlight this issue, with much larger proportions of the population exposed to mental health diminishing financial problems. As we argue above, there is likely to be a need to consider the financial strain that people face day-to-day, perhaps through examining reform to lending criteria and other measures to reduce the cost of living, particularly in housing. Beyond that, the evidence presented here supports suggestions that cash transfer schemes, such as UBI, can function effectively as an upstream intervention. Not only does income affect mental health, decreases in income are particularly harmful. The security and predictability of an unconditional cash transfer would mitigate that impact, ensuring that individuals are protected from a key source of morbidity. Given the rigidity of social gradients within the UK, UBI also offers scope for personal investment in skill development and educational attainment by which to enhance income and independence further, compounding mitigation of social determinants in the process. Ironically, it is this form of transformative policy that would create the conditions under which health-to-income explanations of causality might gain validity.

### Gender

In line with other studies on the subject of mental health during adolescence, we found that young women and girls had a much greater likelihood on average of having depressive symptoms [1]. Importantly, however, the patterns observed in terms of inequalities based on income quintiles were consistent between both males and females. While there may be non-income-based drivers of such inequalities by gender at this age, it remains important to both groups to understand and address economic factors.

## Conclusion

This article presents complex analysis of the relationship between socioeconomic status, including income, and anxiety and depression among 16- to 24-year-olds within Understanding Society. Our within-between fixed effects regression model (Model 2) confirms associations between health and i) between-individual differences in household income, and ii) within-individual variations in household income across time. Both are positive and highly significant predictors of mental health for individuals between 16 and 24 years of age. In contrast to previous reviews, the data presented here facilitates microsimulation modelling of impact on anxiety and depression from changes in socioeconomic circumstances. This enables a much more detailed and complete understanding of the types of socioeconomic intervention, including welfare systems like UBI, that might begin to address some of the causes of adolescent mental illness. The findings provide qualified support for Thomson et al.’s [19] policy recommendations. Although respondents with lower average income have the potential to benefit most from increases, there may be diminishing marginal effects of income on mental health in the absence of progressive fiscal and lending reform. Broader analysis indicates that it is likely that the range of transitory factors particular to adolescents’ lives, including education, work and relationships, mean that focusing solely on those with the lowest incomes is likely to neglect those for whom the impact may be significant and long-standing, such as those who have started work young and those who are single. Analysis using the same models but with older age groups should be undertaken to understand whether associations change through the life course.

## Supporting information

S1 Table List of variables. (DOCX)

S1 Data (DO)
