# Supplementary material for: Understanding the relationship between income and mental health among 16- to 24-year-olds: Analysis of 10 waves (2009–2020) of Understanding Society to enable modelling of income interventions
Source: PLoS One. 2023 Feb 28;18(2):e0279845. doi: 10.1371/journal.pone.0279845 (PMC9974116; doi:10.1371/journal.pone.0279845)
Supplement: S1 Table — (DOCX) [file pone.0279845.s001.docx]

Table S1: List of variables

| **Household level data (UKHLS dataset: hhresp)** |
| --- |
| `w'_hidp |
| `w'_fihhmnnet1_dv |
| `w'_hsrooms |
| `w'_hsbeds |
| `w'_tenure_dv |
| `w'_ieqmoecd_dv |
| `w'_fihhmnnet1_dv |
| `w'_hhsize |
| `w'_nchoecd_dv |
| **Individual level data (UKHLS dataset: indresp)** |
| pidp |
| `w'_hidp |
| `w'_buno_dv |
| `w'_fimnnet_dv |
| `w'_age_dv |
| `w'_sf12mcs_dv |
| `w'_sf12pcs_dv |
| `w'_intdatd_dv |
| `w'_intdatm_dv |
| `w'_sf12mcs_dv |
| `w'_sf12pcs_dv |
| `w'_doby_dv |
| `w'_intdatd_dv |
| `w'_intdatm_dv |
| `w'_intdaty_dv |
| `w'_fenow |
| `w'_gor_dv |
| `w'_hiqual_dv |
| `w'_health |
| `w'_racel_dv |
| `w'_jbstat |
| `w'_mlstat |
| `w'_scghq1_dv |
| `w'_scghq2_dv |
| `w'_school |
| `w'_sex |
| `w'_urban_dv |
| `w'_jlseg_dv |
| `w'_jbseg_dv |
| `w'_jbnssec8_dv |
| `w'_sf12pcs_dv |
| `w'_sf12mcs_dv |
| `w'_jlnssec8_dv |
| `w'_panssec8_dv |
| `w'_mastat_dv |
| `w'_jbhad |
| `w'_scghq1_dv |
| `w'_scghq2_dv |
| `w'_intdaty_dv |
| `w'_nchild_dv |
| `w'_pno |
| `w'_ivfio |
| `w'_saved |
| **All members of household (UKHLS dataset: indall)** |
| pidp |
| `w'_hidp |
| `w'_buno_dv |
| `w'_age_dv |
| `w'_ivfio |
